# Once-weekly erythropoiesis-stimulating agent dosing and haemoglobin outcomes in maintenance haemodialysis: a single-centre evaluation

**DOI:** 10.1093/ckj/sfag334

**Published:** 2026-09-24

**Authors:** Chen Isaac Tseng, Ayushi Gupta, Mala Murugesan, Raji Srinivasan, Tanaji Dasgupta, Packiam Shenbagaraman, Sion Williams

**Affiliations:** Oxford Kidney Unit, Churchill Hospital, Oxford, OX3 7LE, United Kingdom; Research Department of Practice and Policy, School of Pharmacy, University College London, London, WC1H 9JP, United Kingdom; Oxford Kidney Unit, Churchill Hospital, Oxford, OX3 7LE, United Kingdom; Renal and Transplant Centre, Imperial College Healthcare NHS Trust, London, W12 0HS, United Kingdom; Oxford Kidney Unit, Churchill Hospital, Oxford, OX3 7LE, United Kingdom; Milton Keynes University Hospital NHS Foundation Trust, Milton Keynes, MK6 5LD, United Kingdom; Oxford Kidney Unit, Churchill Hospital, Oxford, OX3 7LE, United Kingdom; Great Western Hospital NHS Foundation Trust, Swindon, SN3 6BB, United Kingdom; Oxford Kidney Unit, Churchill Hospital, Oxford, OX3 7LE, United Kingdom; Great Western Hospital NHS Foundation Trust, Swindon, SN3 6BB, United Kingdom; Oxford Kidney Unit, Churchill Hospital, Oxford, OX3 7LE, United Kingdom; Milton Keynes University Hospital NHS Foundation Trust, Milton Keynes, MK6 5LD, United Kingdom; Oxford Kidney Unit, Churchill Hospital, Oxford, OX3 7LE, United Kingdom; Women’s Centre, John Radcliffe Hospital, Oxford, OX3 9DU, United Kingdom

To the Editor,

Erythropoiesis-stimulating agents (ESAs) are central to anaemia management in individuals with end-stage kidney disease undergoing maintenance haemodialysis [1]. In the UK, short-acting ESAs are typically administered thrice weekly, which requires substantial nursing time, and carries environmental and economic costs [2]. With increasing emphasis on sustainable healthcare, including Green Nephrology, evidence on the effectiveness of once-weekly short-acting ESA remains limited [3, 4]. The primary aim of this evaluation was to assess epoetin zeta (Retacrit) effectiveness following a dosing frequency change from thrice weekly to once weekly in a prevalent haemodialysis cohort.

A retrospective analysis was conducted using data from the Cerner electronic patient record at two haemodialysis units within the Oxford Kidney Unit. This evaluation adhered to the Caldicott principles and UK Health Research Authority guidance. Ethics approval was not required.

All adults (*n* = 111) across two haemodialysis units were reviewed in multidisciplinary meetings for the suitability of ESA frequency change. Exclusion criteria applied to those remaining on thrice-weekly ESA (*n* = 25), already on once-weekly ESA (*n* = 19), hospitalisation (*n* = 8), transplantation (*n* = 6), declined ESA frequency change (*n* = 1), not receiving ESA (*n* = 4), surgery (*n* = 2), holiday (*n* = 1), death (*n* = 3), receiving vadadustat (*n* = 2), and ESA frequency reverted back to twice weekly or thrice weekly (*n* = 2).

Baseline data on demographics, dialysis frequency, haemoglobin (Hb), ferritin level, ESA dosing, and total weekly ESA requirement were summarised using descriptive statistics (Fig. 1). The primary outcome was Hb response after ESA frequency change, analysed using a linear mixed-effects model. Estimated mean Hb at 1-month follow-up and that at 2-month follow-up were compared with baseline; a non-inferiority margin of -5 g/l was applied to reflect a threshold beyond which Hb decline was considered clinically meaningful and likely to prompt dose change in practice. The secondary outcome used the Wilcoxon signed-rank test to assess whether the frequency change affected the total weekly ESA requirement. Statistical significance was set at a two-sided *P* < .05. Analyses were performed using Stata version 18.

Thirty-eight individuals were included (mean age 63 years, 65.8% male, 63.2% White). Most underwent thrice-weekly dialysis (*n* = 33). Median baseline Hb was 111 g/l (interquartile range [IQR] 106–120) and weekly ESA dose of 13 500 units (IQR 12 000–20 000). Estimated mean Hb was 110 g/l (95% CI 107–114) at baseline, 111 g/l (95% CI 107–115) at the intervention month, 110 g/l (95% CI 107–114) at 1-month follow-up, and 109 g/l (95% CI 105–112) at 2-month follow-up. Compared with baseline, mean differences were -0.4 g/l (95% CI -3.9 to 3.1; *P* = .802) at 1-month follow-up and -2.0 g/l (95% CI -5.5 to 1.5; *P* = 0.257) at 2-month follow-up. Hb control met the non-inferiority threshold at 1-month follow-up, but not at 2-month follow-up. Weekly ESA requirements remained stable; no significant differences were observed between baseline and 1-month (*P* = .98) or 2-month (*P* = .48) follow-up. Notably, this practice change reduced syringe use by ∼32 per month in this cohort.

To our knowledge, this is the first real-world evaluation of short-acting ESA dosing frequency optimisation in haemodialysis. These preliminary findings suggest that once-weekly ESA dosing may maintain Hb control comparably to thrice-weekly dosing, although non-inferiority was not sustained at 2-month follow-up. Given the small sample size, residual confounding, and risk of selection bias from high drop-out, results warrant cautious interpretation. Nonetheless, the implications may extend beyond clinical effectiveness to reduced nursing workload, less pharmaceutical waste, and more sustainable dialysis care [5]. Larger studies with longer follow-up are needed to determine the maximal safe and effective short-acting ESA that may be administered weekly in haemodialysis, identify individuals less suited to this dosing strategy, and confirm the cost-effectiveness and environmental impact of this prescribing practice.

**Figure 1:**
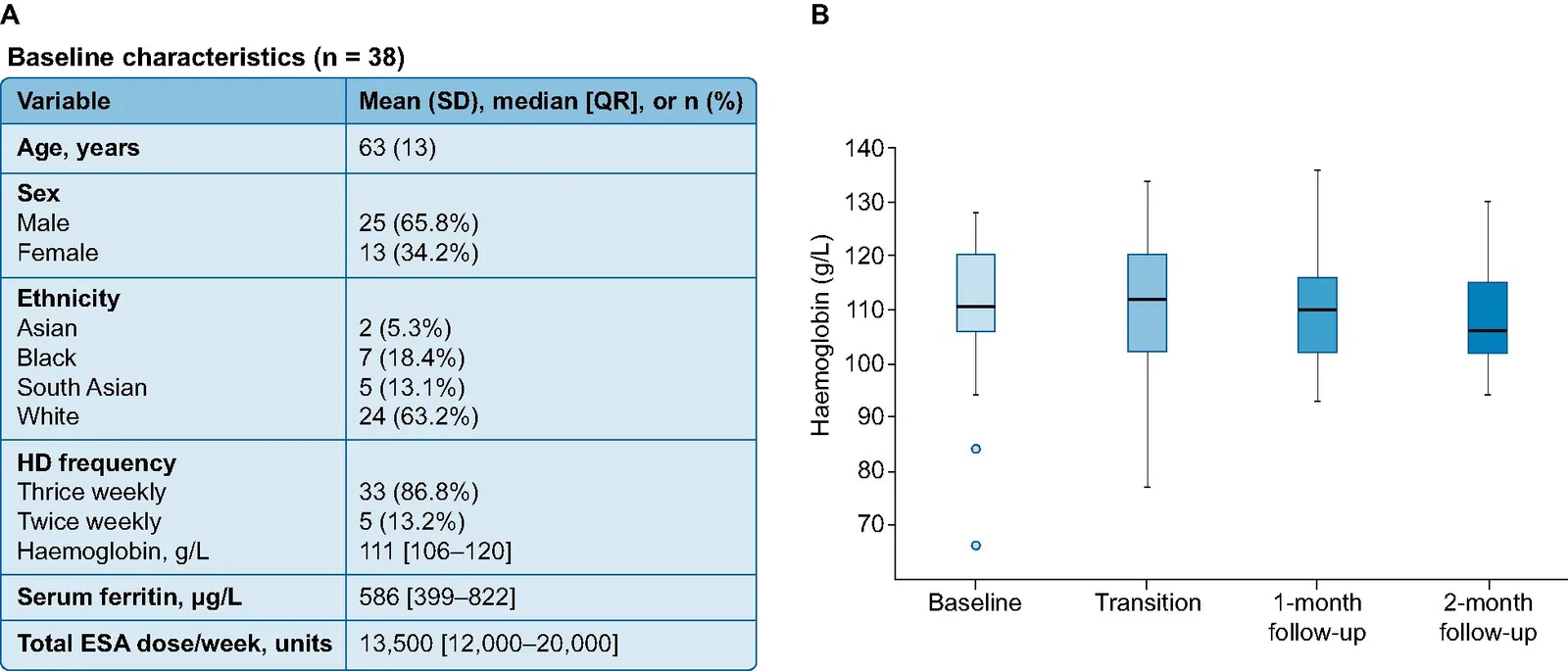
(A) Demographic information and clinical characteristics of the study cohort. (B) Boxplot of Hb levels at baseline, transition (ESA frequency reduced from thrice to once weekly; total weekly dose unchanged), and one- and two-month follow-ups. Boxes represent medians with IQRs, whiskers denote minimum and maximum values within 1.5 times of IQR, and dots indicate outliers.
